# Reconstitution of rat fetal testis during the masculinisation programming window induces focal dysgenesis consistent with testicular dysgenesis syndrome

**DOI:** 10.1038/s41598-020-75803-1

**Published:** 2020-11-04

**Authors:** Ellie Smart, Joni Macdonald, Lee B. Smith, Rod T. Mitchell

**Affiliations:** 1 MRC Centre for Reproductive Health, The Queen’s Medical Research Institute, The University of Edinburgh, 47 Little France Crescent, Edinburgh, EH16 4TJ Scotland, UK; 2 Faculty of Science, University of Newcastle, Callaghan, Australia; 3 Edinburgh Royal Hospital for Sick Children, 9 Sciennes Road, Edinburgh, EH9 1LF Scotland, UK

**Keywords:** Reproductive biology, Reproductive disorders

## Abstract

Focal dysgenesis is a consistent feature of testicular dysgenesis syndrome (TDS) in humans. Rodent studies show that perturbation of androgens (e.g. following phthalate exposure) during a fetal masculinisation programming window (MPW) predisposes to a TDS phenotype. This study aimed to determine whether dissociation and reconstitution of rat fetal testis tissue during the MPW can be used to model and manipulate seminiferous cord development, including induction of focal dysgenesis, as described in TDS. Dissociated fetal rat testes were xenotransplanted subcutaneously into recipient mice for 4 weeks. Transplanted mice were treated with vehicle or di-*n*-butyl-phthalate (DBP, a plasticising chemical known to induce testicular dysgenesis in vivo in rats). Testosterone production by the transplants was measured in recipient mice and immunofluorescence was performed on the retrieved transplants to identify features consistent with focal testicular dysgenesis. Re-aggregation of rat fetal testis tissue xenotransplants during the MPW results in reconstitution of seminiferous cords. Features of focal testicular dysgenesis were present in re-aggregated testis, including ectopic Sertoli cells and intratubular Leydig cells (ITLCs). DBP exposure of recipient mice reduced androgen-dependent seminal vesicle weight (8.3 vs 26.7 mg; p < 0.05), but did not enhance features of focal dysgenesis including number of ITLCs (0.07 vs 0.10 cells/mm^2^; p > 0.05). We conclude that seminiferous cord reformation during the MPW results in development of focal dysgenesis. The system may be used to separate specific effects (e.g. androgen suppression) of individual chemical exposures from other mechanisms that may be conserved in TDS.

## Introduction

A testicular dysgenesis syndrome (TDS) has been proposed which describes a group of associated disorders of the male reproductive tract with a common origin in fetal life^1,2^. In humans, this includes hypospadias, cryptorchidism, testicular germ cell cancer, hypogonadism and low sperm counts. The incidence of these disorders has been increasing over recent decades^3^. These disorders are often associated with (focal) dysgenetic changes in the testis including abnormally shaped seminiferous tubules, Sertoli-cell-only cords and Leydig cell nodules^4,5^.

TDS is believed to result from impaired androgen production or action in utero^6^, and a masculinisation programming window (MPW) has been identified during which androgen action programmes the subsequent development and function of the entire male reproductive tract^7–9^. In rats, the MPW has been defined as embryonic day (e) 15.5–18.5, during which reduction in androgen production or action leads to the development of TDS^8,9^, whilst in humans it is believed to be between 8–12 weeks gestation^8–10^. A TDS-like syndrome can be induced in fetal rats by exposing the mother to Di-*n*-butyl phthalate (DBP), a plasticising chemical, only during the MPW, resulting in cryptorchidism, hypospadias and focal dysgenesis within the testis even though the DBP exposure occurs after gonad formation and early development^11^. Focal dysgenesis consists of malformed seminiferous cords with reduction or absence of germ cells and presence of intratubular Leydig cells^12,13^. Dysgenetic areas also include central aggregation of fetal Leydig cells at e17.5 and ectopic Sertoli cells in 80% of cases by e21.5^8^. These ectopic Sertoli cells have been shown to result from breakdown of previously formed seminiferous cords rather than de novo differentiation^14^.

The rationale for the study was based on the fact that current experimental models to investigate the development of focal testicular dysgenesis rely on high-dose exposure to chemicals that can affect multiple signalling pathways and/or result in toxicity. Therefore, a model system that induces features of focal testicular dysgenesis and does not involve chemical exposure may represent a more tractable model for determining mechanisms underlying the pathogenesis of testicular dysgenesis in men with testicular disorders.

We hypothesised that a re-aggregation model, involving testicular dissociation and reconstitution can be applied to the rat fetal testis during a critical period (MPW) of fetal development, and that this approach may be used to model and manipulate seminiferous cord development, including induction of focal dysgenesis, as described in patients with common male reproductive disorders (often referred to as TDS disorders)^14^.

## Methods

### Animals

Ethical approval for animal studies was obtained from University of Edinburgh’s Local Ethical Review Committee and animal studies were conducted according to UK Home Office guidelines under Project License (PPL 60/4564). Time-mated pregnant female Wistar rats (Harlan UK) were used to obtain fetal tissues. Rats were housed for a minimum of 2 weeks prior to use in experimental studies and had free access to food and water. Housing conditions consisted of: lights 0700–1900 h, temperature 19–21 ∘C and humidity 45–65%. Pregnant rats were culled by CO_2_ inhalation followed by cervical dislocation. Fetuses were removed, decapitated and placed in ice-cold PBS (Sigma-Aldrich).

### Testis tissue dissociation

Testes from embryonic day (e)17.5 Wistar rats (n = 40 from 4 separate litters) were dissected and digested in 1 mg/ml Collagenase IV (Sigma, UK) followed by 10 mg/ml DNase 1 (Sigma) diluted in Hank’s balanced salt solution (HBSS, Gibco, UK). Cells were centrifuged and the supernatant removed before being re-suspended in 10 ml of media (L15 Liebowitz Media, Sigma) and transferred through a 70 μm mesh filter to ensure only single cells were present.

### Xenotransplantation and treatments

Cell suspensions (10^6^ cells) were placed in 0.5 ml of extracellular matrix gel (Matrigel, BD Biosciences, US) and injected subcutaneously (termed ‘re-aggregated xenotransplant’) into castrate immunocompromised CD-1 nude mice (n = 6; Charles River, UK). Cell suspensions (n = 6 per mouse) were inserted and host mice (n = 3 per treatment) were treated with vehicle (corn oil) or DBP (Sigma, 500 mg/kg/day) in corn oil daily by oral gavage, from day 5 after transplantation. After 4 weeks, mice were killed by cervical dislocation and all transplants retrieved and fixed in Bouins Solution for 6 h. Transplants were transferred to 70% ethanol and fixed in paraffin using standard procedures. Additional controls for the transplantation procedure consisted of intact (non-dissociated) rat fetal (e17.5) testis tissue transplants (termed ‘tissue xenografts’) that were grafted for 7 days as previously described^15^.

### Haematoxylin and eosin staining

Tissue sections (5 μm thickness) were placed onto glass slides before being de-waxed in xylene and rehydrated using decreasing concentrations of ethanol. Slides were stained with Haematoxylin for 2.5 min before being placed in Scott’s tap water for 30 s. Slides were submerged in Eosin for 10 s before being dehydrated in increasing concentrations of ethanol. Slides were then placed in xylene before mounting with Pertex (Cell path, UK).

### Immunofluorescence

Antibodies and concentrations used for immunofluorescence are described in Table 1. Sections were de-waxed in xylene and rehydrated using decreasing concentrations of ethanol. Washes in Tris buffered saline occurred between each subsequent stage. Antigen retrieval was performed in 0.1 M citrate buffer (pH 6) followed by an endogenous peroxidase block of 3% (v/v) H_2_O_2_ in methanol for 30 min. Sections were incubated in normal horse serum diluted 1:5 with TBS containing 5%(w/v) bovine serum albumin (BSA) for 30 min before the primary antibody diluted in chicken serum was added and left overnight at 4 °C in a humidified chamber. Sections were incubated with the appropriate secondary antibody diluted at 1:200 in normal chicken serum for 30 min. Sections were kept in the dark for all further incubations. Tyramide signal amplification kit (Perkin Elmer, UK) was used as the visualisation reagent at 1:50 for 10 min. Slides were placed in citrate buffer and microwaved for 2.5 min. Sections were left to cool for 30 min and then incubated in normal chicken serum, primary antibody, secondary antibody and Tyramide as described above. The process was repeated a third time for triple immunofluorescence. Sections were counterstained with 4,6-diamino-2-phenylindole (DAPI, Sigma) diluted in TBS at 1:750 for 10 min. Slides were mounted with Permafluor (Thermo Scientific, UK). Images were captured using a LSM 710 Confocal microscope (Carl Zeiss, Hertfordshire, UK).

**Table 1:** Antibodies used for immunofluorescence.

Antibody	Cell type (s)	Species	Dilution	Source	Secondary antibody
SOX9	Sertoli cells	Rabbit	1:500	Chemicon^a^	Chicken anti-rabbit peroxidase^c^
MVH	Germ cells	Rabbit	1:400	Abcam^b^	Chicken anti-rabbit peroxidase^c^
3β-HSD	Leydig cell (steroidogenesis)	Goat	1:400	Santa Cruz^c^	Chicken anti-goat peroxidase^c^
SMA	Peritubular cells	Mouse	1:800	Sigma^d^	Chicken anti-mouse peroxidase^c^

Primary antibodies were applied in sequence according to this table.

^a^Chemicon International, CA, UK.

^b^Abcam, Cambridge, UK.

^c^SantaCruz Biotechnology, CA, USA.

^d^Sigma, Poole, UK.

### Determination of tubular diameter

Stereological analysis was performed using Image pro-Plus software. This was used to select 25 random fields per section for measurement. In each field of view the diameter of all the seminiferous cords was measured and averaged for each section, with a minimum of 90 cords measured per section (n = 3 per group). Differences between e17.5, and re-aggregated xenotransplants were examined using Student’s t-test followed by Bonferonni post hoc test using GraphPad Prism 5 (GraphPad Software Inc.). Statistical significance was set at p < 0.05.

### Quantification of intratubular Leydig cells

Two tiles per section were selected using SOX9 (Sertoli cell) channel to avoid bias when selecting sections. For each 2 by 2 tile the number of 3β-HSD positive cells within re-formed seminiferous cords was counted. In total, five transplants from three different host mice were examined per treatment group. Images were captured using an LSM 510 Meta Confocal microscope (Carl Zeiss, Hertfordshire, UK). The number of 3β-HSD positive cells are expressed relative to the percentage of the tile that was occupied by seminiferous cords. Normally distributed data was analysed using one-way ANOVA or Student’s t-test, whilst non-parametric data was analysed using the Mann–Whitney test. All statistical analyses was performed using GraphPad Prism 8 (GraphPad Software Inc.) and statistical significance was set at p < 0.05.

## Results

### Reconstitution of seminiferous cords in re-aggregated rat fetal testis cells xenografts

**Figure 1:**
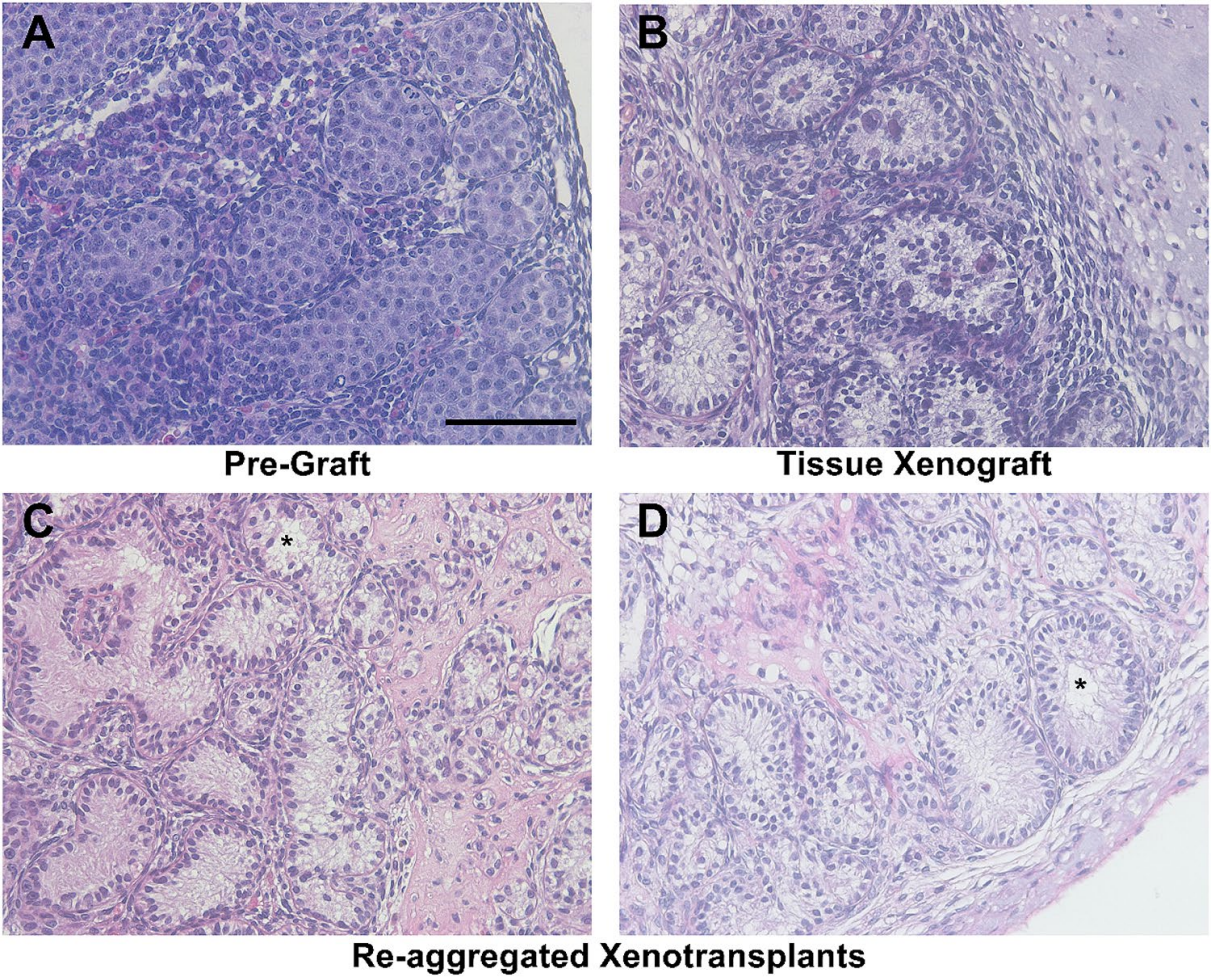
Re-aggregated fetal rat testis xenotransplants form seminiferous tubules. Haematoxylin and Eosin staining of rat fetal testis, tissue xenotransplants and re-aggregated xenotransplants. **(A)** Pre-transplant control testis (e17.5). **(B)** Whole rat fetal testis tissues xenotransplanted for 4 weeks. **(C,D)** Re-aggregated rat fetal testis xenotransplants 4 weeks after transplanting. Images from re-aggregated transplants are representative of 6 different transplants from 3 different ‘vehicle exposed’ recipient mice. Scale bar = 100 μm. * indicates areas of lumen formation.

The general morphology of the re-aggregated fetal rat testis tissue was assessed by haematoxylin and eosin staining and compared with pre-graft control and whole tissue xenotransplants (Fig. 1).

**Figure 2:**
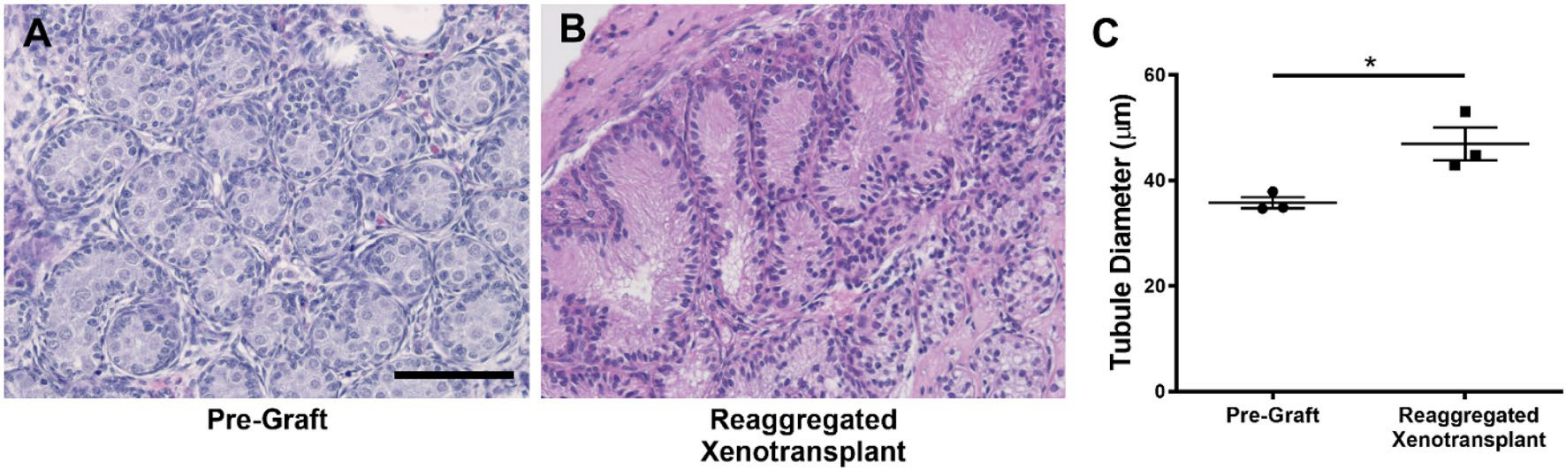
Increased tubular diameter in re-aggregated fetal rat testis xenotransplants. **(A)** Pre-transplant control testis at e17.5. **(B)** Re-aggregated xenotransplant with evidence of luminal development. **(C)** Quantification of seminiferous tubule diameter in re-aggregated testis tissue xenotransplants compared with pre-transplant control. Statistical analysis performed using students t-test. Values are mean ± SEM per group for n = 3 *p < 0.05. Scale bar = 100 μm.

In e17.5 pre-graft controls, seminiferous cords consisted of tightly packed cells with no apparent lumen (Fig. 1A). Whole tissue xenotransplants exhibited retention of seminiferous cords with a reduction in cell density (Fig. 1B). The re-aggregated xenotransplants formed seminiferous cords of differing size with a reduction in cell density compared with pre-graft control, coinciding with lumen formation in the centre of some of the cords (Fig. 1C,D). Seminiferous cord/tubule diameter was measured in reaggregated tissue xenotransplants and compared to pre-graft (e17.5) control (Fig. 2). In re-aggregated testicular xenotransplants, tubular diameter was significantly increased (46.9 vs 35.8 µm, p < 0.05) compared to pre-graft controls.

**Figure 3:**
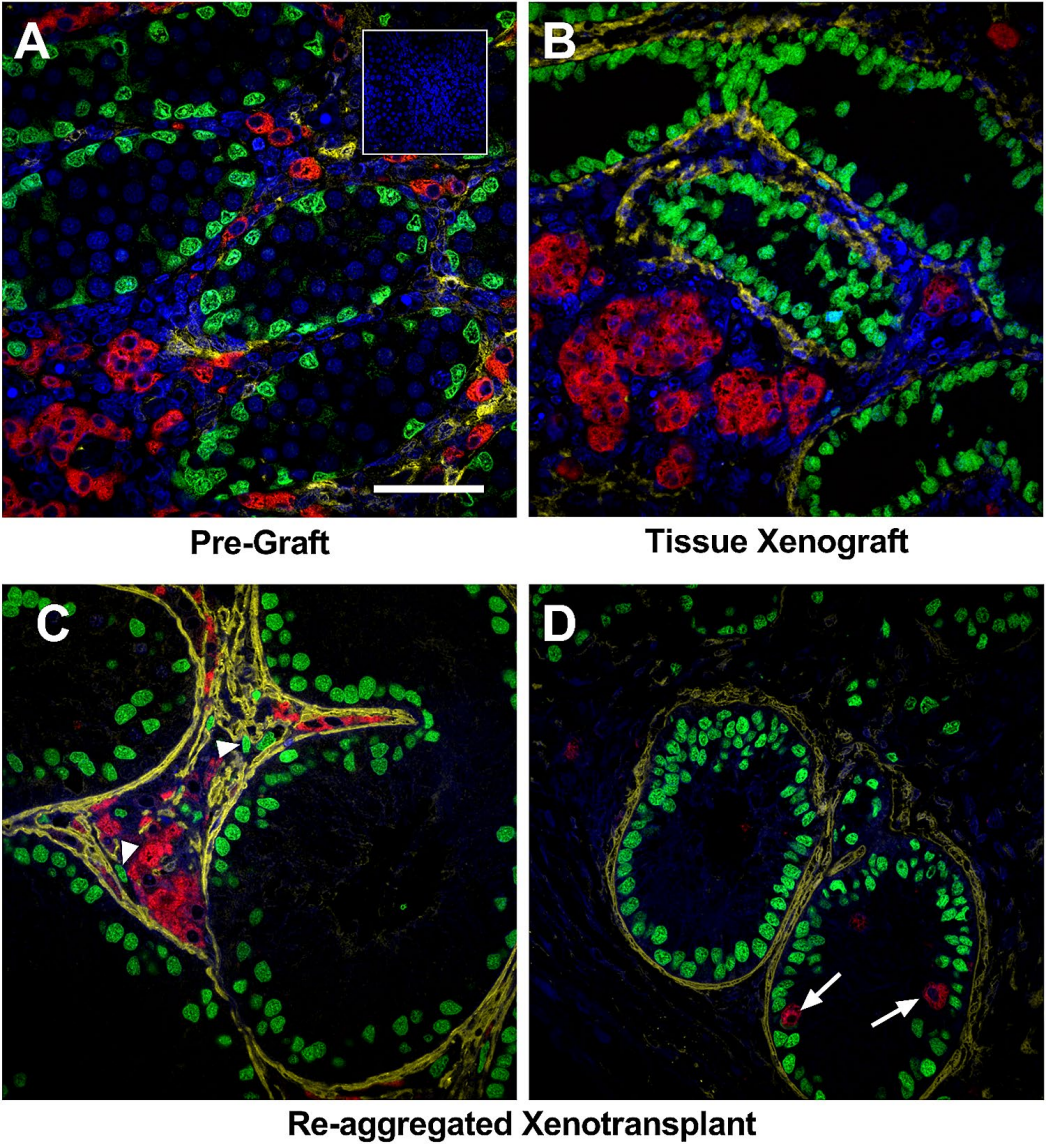
Re-aggregated rat fetal testis xenotransplants display development of the basement membrane. Triple immunofluorescence for SMA (yellow; peritubular myoid cells), SOX9 (green; Sertoli cells) and 3β-HSD (red: Leydig cells). **(A)** Pre-transplant control testis at e17.5. **(B)** Whole tissue xenotransplant. **(C)** Re-aggregated xenotransplant with ectopic Sertoli cells (arrowheads). **(D)** Re-aggregated xenotransplant with intratubular Leydig cells (arrows). Scale bar = 100 μm. Inset—negative control.

Immunofluorescence was performed to compare the development of the seminiferous cords and interstitial compartment between whole tissue and re-aggregated xenotransplants (Fig. 3). Smooth muscle actin (SMA) was used as a marker of peritubular myoid cells which are involved in development of the basement membrane of the seminiferous cords. In pre-graft tissues, seminiferous cords containing Sertoli cells were distinct from the interstitium; however, there was limited SMA expression in peritubular cells (Fig. 3A). Expression of SMA was present in the basal region of whole tissue xenografts (Fig. 3B), demonstrating peritubular myoid cell maturation and presumptive development of the basement membrane in rat fetal testis xenografts. A similar pattern of SMA expression to that of the whole tissue xenografts was also seen in re-aggregated xenotransplants (Fig. 3C). In addition, the interstitium of the tissue and re-aggregated xenotransplants contained abundant Leydig cells and occasional ectopic Sertoli cells (Fig. 3B,C). Leydig cells were also identified within the seminiferous cords in a proportion of xenotransplants (Fig. 3D). These intratubular Leydig cells (ITLCs) appeared to be restricted to cords that were Sertoli cell only. Taken together, these results demonstrate that dissociated rat fetal testis tissue xenotransplants can reform seminiferous cords with evidence of preservation of the two major compartments and that the reconsituted testes display features consistent with focal dysgenesis.

### Germ cells are present within the seminiferous cords of re-aggregated rat fetal testis tissues

**Figure 4:**
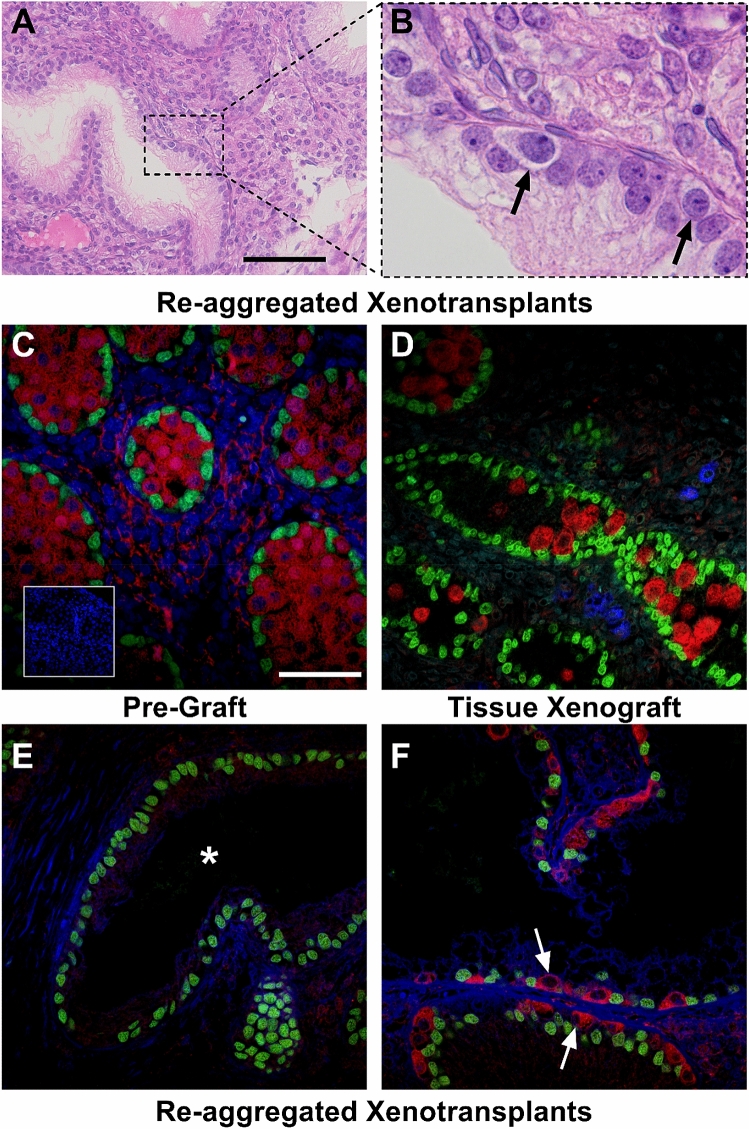
Germ cells are present in re-aggregated rat fetal testis xenotransplants. **(A)** Haematoxylin and eosin staining of re-aggregated rat fetal testis xenotransplant. Scale bar = 100 μm. **(B)** High magnification (× 100) of area highlighted in **(A)** showing presence of cells with germ cell morphology (arrows). **(C–F)** Double immunofluorescence for MVH (red; germ cells) and SOX9 (green; Sertoli cells). **(C)** Germ cells (MVH^+^) are densely packed within the seminiferous cords in e17.5 testis pre-transplant control. Scale bar = 50 µm. Inset shows corresponding negative control. **(D)** Whole tissue rat fetal testis xenotransplant with germ cells present within the majority of seminiferous cords. **(E)** Re-aggregated rat fetal testis xenotransplant with Sertoli cell only (SOX9^+^) appearance. **(F)** Re-aggregated rat fetal testis xenotransplant with MVH^+^ germ cells (arrows) located within the seminiferous tubule.

Analysis of the haematoxylin and eosin stained sections identified infrequent cells with germ cell morphology in re-aggregated fetal testis tissues (Fig. 4A,B). These germ cells were located at the base of the seminiferous cords (Fig. 4B). To further characterise these cells we performed double immunofluorescence staining for MVH (germ cells) and SOX9 (Sertoli cells). We identified MVH expressing germ cells in the pre-graft e17.5 control tissue (Fig. 4C). These large round cells were located in the centre of the seminiferous cords consistent with gonocytes that are known to represent the germ cell population at e17.5^16^. Germ cells were also present in all xenografts derived from whole rat fetal testis tissue, although they appeared reduced in number in the whole tissue xenograft compared to the pre-graft control tissues (Fig. 4D). These cells were present in the centre of the cord as well as adjacent to the basement membrane indicating their transition from gonocyte to spermatogonia. The majority (80%) of re-aggregated rat fetal testis tissue xenografts did not contain MVH-expressing (germ) cells and were therefore considered Sertoli cell only (Fig. 4E). However, MVH-expressing germ cells were identified in 20% of re-aggregated xenografts and these germ cells were all located adjacent to the basement membrane of the seminiferous cords (Fig. 4F). Where present, these germ cells were located adjacent to the basement membrane and had a flattened appearance typical of spermatogonia.

### Effect of DBP exposure on seminiferous cord formation in re-aggregated rat fetal testis xenotransplants

**Figure 5:**
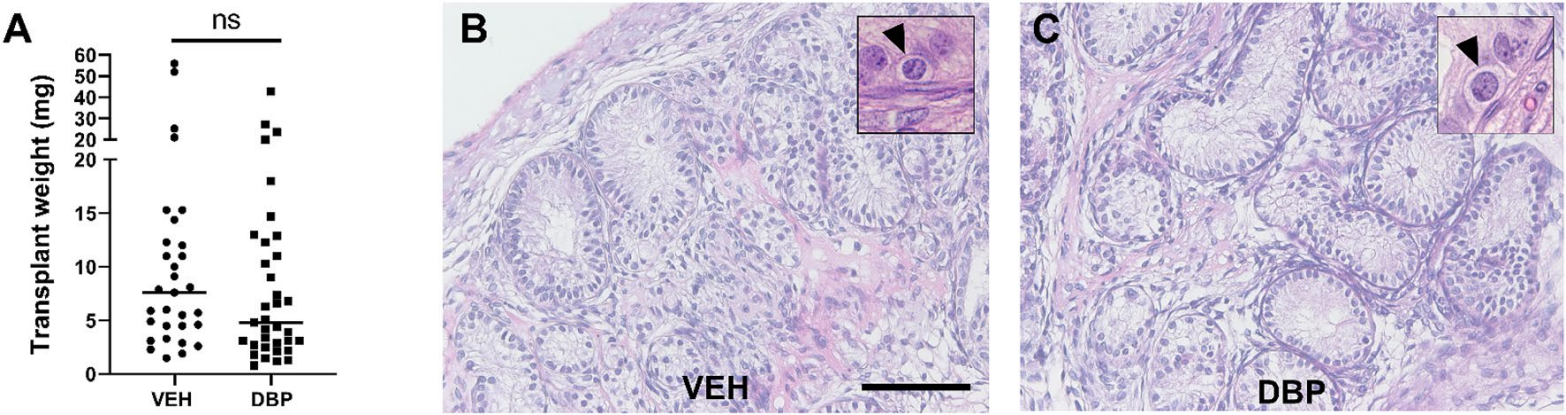
DBP exposure does not prevent re-formation of testicular structure in reaggregated rat fetal testis xenotransplants. **(A)** Transplant weights from re-aggregated rat fetal testis xenotransplants obtained from host-mice (n = 5–6) exposed to vehicle (VEH) control or DBP. Each data point represents an individual xenotransplant. Statistical analysis performed using Mann–Whitney test. *ns* non-significant. Bar represents median value. **(B,C)** Haematoxylin and eosin staining of xenotransplants exposed to **(B)** vehicle or **(C)** DBP. Scale bar = 100 µm. Insets highlight germ cells (arrowheads).

To determine if DBP exposure affected re-aggregation of rat fetal testis tissue xenotransplants, host mice were treated with either DBP or vehicle control for 4 weeks, commencing 5 days after transplantation (Fig. 5). Total graft weight was not significantly different between control and DBP-exposed groups indicating similar growth of the xenografts (Fig. 5A). Overall structure and morphology of the re-formed testicular tissues was also similar in re-aggregated rat fetal testis xenografts from vehicle- and DBP-exposed host mice and seminiferous cord formation was clearly evident in grafts from both groups (Fig. 5 B,C). Occasional germ cells were also present in re-aggregated tissues from some of the DBP- and vehicle-exposed xenografts (Fig. 5 B,C).

### Effect of DBP exposure on Leydig cell function in re-aggregated rat fetal testis xenotransplants

**Figure 6:**
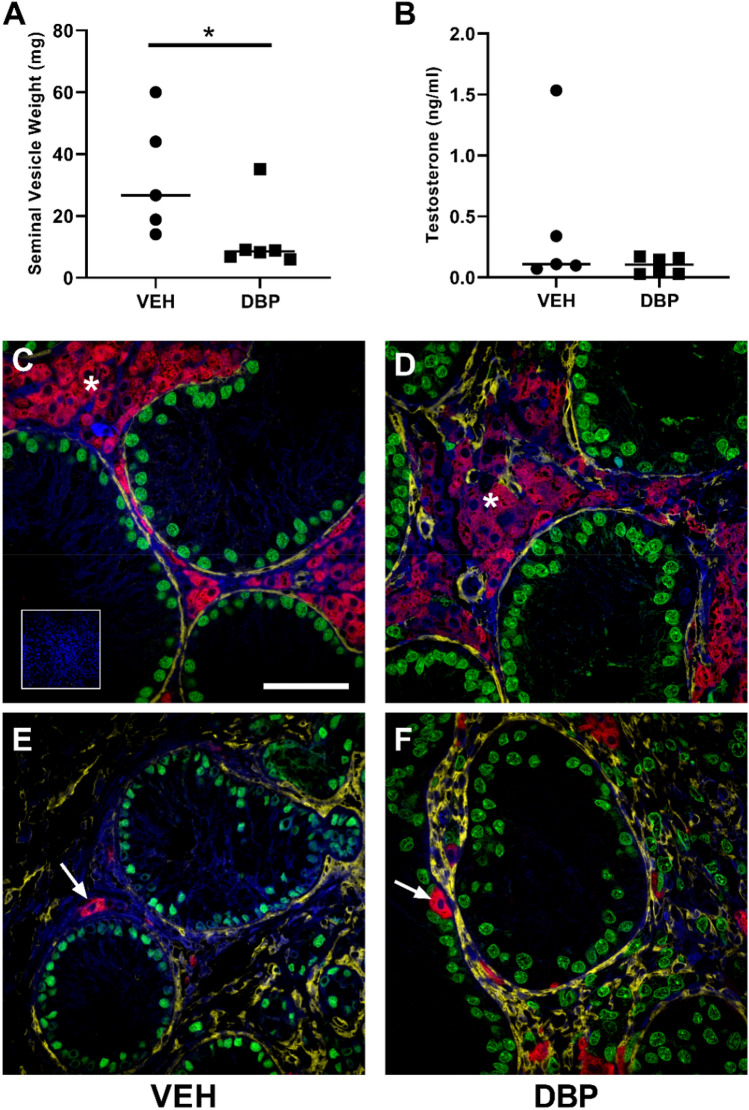
Leydig cells are present in re-aggregated rat fetal testis xenotransplants although seminal vesicle weight is reduced in host mice exposed to DBP. **(A)** Seminal vesicle weight and **(B)** serum testosterone in xenotransplanted host mice (n = 5–6) exposed to vehicle (VEH) control or DBP. Each data point represents total transplant weight obtained from a single host mouse. Statistical analysis performed using Mann–Whitney test. Bar represents median value. * p < 0.05. **(C–F)** Triple immunofluorescence for SMA (yellow; basement membrane) and SOX9 (green; Sertoli cells) and 3β-HSD (red: Leydig cells) in vehicle- (VEH) and DBP-exposed xenotransplants, demonstrating Leydig cell aggregates (*) and individual Leydig cells (arrows). Scale bar = 50 µm.

Leydig cell testosterone production was assessed by measuring seminal vesicle weight (androgen-dependent organ) and serum testosterone in host mice. Seminal vesicle weight was significantly reduced in DBP-exposed host mice compared with vehicle-exposed controls (8.3 vs 26.7 mg; p < 0.05) indicating a DBP-induced reduction in xenograft testosterone production over the duration of the grafting period (Fig. 6A), but there was no difference in serum testosterone at a single time-point at the end of the experiment (Fig. 6B). Immunofluorescence for 3β-HSD demonstrated the presence of Leydig cells in the interstitium in both vehicle- and DBP-exposed xenografts (Fig. 6C–F). The density of Leydig cells was variable in both vehicle- and DBP-exposed xenografts with some grafts exhibiting high numbers of densely packed 3β-HSD expressing Leydig cells (Fig. 6C,D), whilst others had individual Leydig cells distributed sparsely throughout the interstitium (Fig. 6E,F).

**Figure 7:**
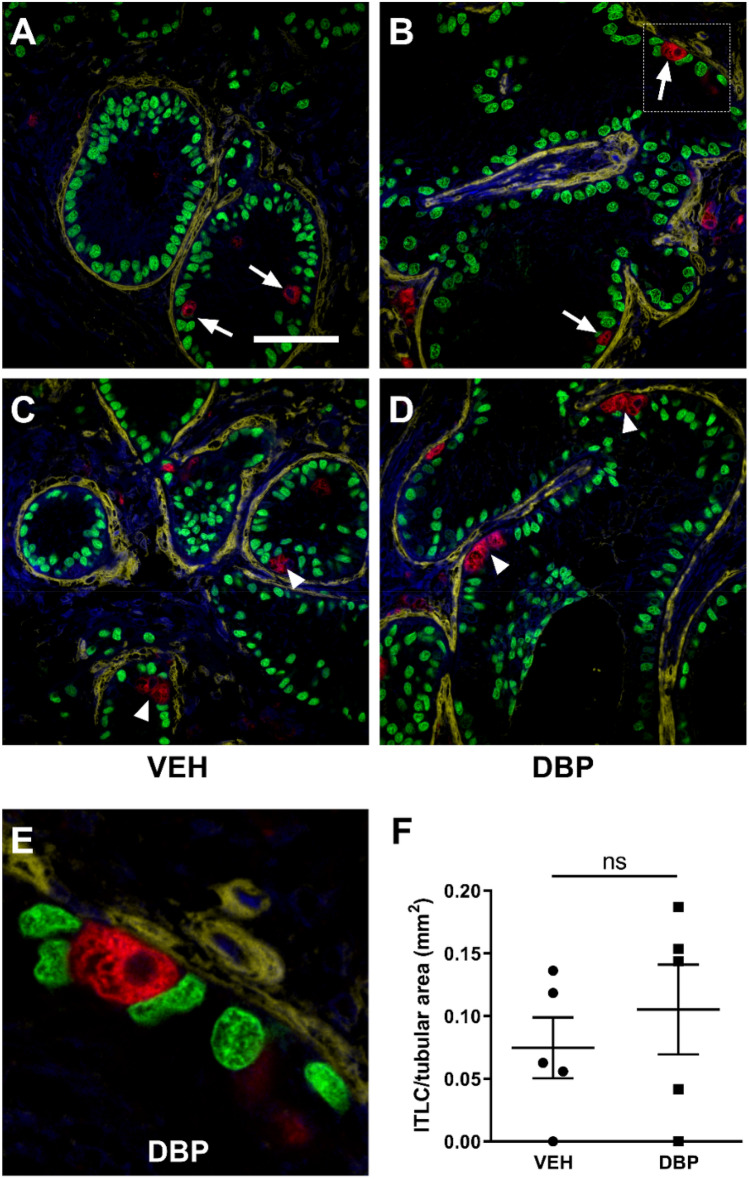
Intratubular Leydig cells are present in re-aggregated rat fetal testis xenotransplants. **(A–D)** Triple immunofluorescence for SMA (yellow; basement membrane) and SOX9 (green; Sertoli cells) and 3β-HSD (red: Leydig cells) in vehicle- (VEH) and DBP-exposed xenotransplants, demonstrating individual ITLCs (arrows; A,B) and ITLC clusters (arrowheads; **C,D**). Scale bar = 50 µm. **(E)** High magnification of area indicated by box in **(D)** showing ITLC. **(F)** ITLCs in xenotransplants from host mice exposed to vehicle (VEH) control or DBP. Each data point represents an individual xenotransplant. Statistical analysis performed using students t-test. Values are mean ± SEM per group for n = 5.

ITLCs were identified in xenografts from vehicle- and DBP-exposed animals (Fig. 7A–E). These consisted mainly of small numbers of single cells within a tubule (Fig. 7A,B). Occasionally a small cluster of ITLCs were seen (Fig. 7C,D). There was no difference in the number of ITLCs in xenografts from DBP-exposed host mice compared with vehicle-exposed controls (0.07 vs 0.10 cells/mm^2^; P > 0.05) (Fig. 7F).

## Discussion

The present study investigated the potential for reconstitution of seminiferous cords after dissociation of rat fetal testes during the MPW, a critical period for fetal testis development. We also assessed the suitability of this approach for manipulation of testicular development and function during the MPW, including the induction of focal testicular dysgenesis.

The results demonstrate that seminiferous cords reform in xenotransplants of dissociated rat fetal testes taken during the MPW. The seminiferous cords contained Sertoli and germ cells and the peritubular myoid cells had begun to express smooth muscle actin indicating functional maturation of this peri-basal cell population. Where present, germ cells were located adjacent to the basement membrane and had a flattened appearance, indicating differentiation of gonocyte to spermatogonia during the xenografting period. Leydig cell function was demonstrated within the reaggregated testis xenografts as indicated by testosterone production and seminal vesicle weight in the host mice. Leydig cell steroidogenesis was also demonstrated by CYP11A1 expression within the xenografts. Previous studies have demonstrated that dissociated testis cells from pnd7 rats can develop spherical aggregate structures in-vitro and that these can develop into tissues with the appearance of seminiferous tubules^17^. Reformation of seminiferous cords has also been demonstrated in xenografts of dissociated testis tissues from rats and mice during late fetal and early postnatal life^18^. Seminiferous cord structures were demonstrated in these xenografts, the majority of which were Sertoli cell-only. However, further analysis of the development and function of the somatic cell populations was not reported in these tissues.

A key aspect of the present study was to determine whether the reaggregation model can be used to disrupt testicular development and function during the MPW, a critical period during which the development of the male reproductive tract is programmed (Welsh et al., 2008). In addition to the reformation of seminiferous cords, we also observed typical features of focal dysgenesis such as Leydig cell aggregates and ectopic Sertoli cells within the reaggregated xenografts^8,14^. Ectopic Sertoli cells were identified in re-aggregated testis xenotransplants and Leydig cell distribution varied from a few isolated cells to large clusters. In addition, ITLCs were present within re-aggregated rat fetal testis xenotransplants. ITLCs and ectopic Sertoli cells have previously been identified in focal dysgenetic areas associated with Leydig cell aggregates and malformed cords in postnatal life after DBP exposure during the MPW^8,14,19^. A common feature between the present study and previous studies involving DBP exposure is that ITLCs were only identified in seminiferous cords/tubules that were devoid of germ cells, suggesting that the presence of ITLCs prevents germ cell survival in cords/tubules or vice versa, which warrants further investigation^14,19^. The present study does not determine whether the seminiferous cords in reaggregated tissues initially form normally and subsequently develop dysgenetic areas, or whether the focal dysgenesis occurs during the reformation process itself. We would hypothesise that it is the latter as we have not previously observed development of dysgenetic areas over time in whole tissue xenografts. Additional studies involving assessment of reaggregated xenografts at earlier timepoints would be required to address this question.

Seminiferous cords in re-aggregated testis tissues primarily contained Sertoli cells, and many cords were Sertoli cell-only. Germ cells were identified by MVH staining and were noted to be infrequent in re-aggregated transplants, similar to results from previous studies using xenografts of dissociated fetal and postnatal rodent testis^17,18^. A subnormal complement of germ cells is also a feature of testicular dysgenesis that occurs in TDS^8^. It should be recognised that whilst there remains some debate about whether TDS represents a distinct clinical entity, the TDS hypothesis provides a useful basis to explore the association of frequently occurring male reproductive disorders and to investigate common mechanisms that predispose to these disorders.

Exposure to DBP did not affect seminiferous cord formation in re-aggregated testis transplants, with similar appearance of re-formed cords in both vehicle- and DBP-exposed xenotransplants. These findings are in keeping with previous studies which indicated that initial seminiferous cord formation was unaffected by DBP exposure^14^. Whilst it is possible that seminiferous cord formation may have initiated prior to commencing DBP treatment of host mice, it has been shown previously that it takes 10 days for cultures of isolated cells from pnd7 rat testis to reform typical cord-like structures composed of Sertoli cells, surrounded by peritubular cells^17^. When cell suspensions were cultured in extracellular matrix, rudimentary cord-like structures were formed by day 3 but typical seminiferous cords were only evident after a further 2 weeks of xenografting^17^. Taken together, we conclude that DBP does not affect initial formation of seminiferous cords in vivo or following reconstitution of testis obtained during the MPW.

Overall, there was a wide variation in transplant size (range 1–56 mg) in both vehicle- and DBP-exposed groups with no difference between groups. These results suggest that DBP does not affect growth of re-aggregated rat fetal testis xenotransplants and that growth is dependent on additional factors e.g. initial graft size, graft location or timing/degree of vascularisation in both treatment groups.

Leydig cell dysfunction is the most frequently reported testicular effect of DBP exposure in rats during fetal life. In the present study, DBP exposure resulted in a significant reduction in seminal vesicle weight in host mice compared to vehicle-exposed controls. Seminal vesicles are androgen-dependent organs and provide a readout of testosterone production over an extended period^20^. Host mice were castrate and are not producing endogenous testosterone, hence the lower seminal vesicle weights in DBP-exposed animals indicates a reduction in testosterone production from re-aggregated xenotransplants. This is despite the low serum testosterone in the majority of animals from both vehicle- and DBP-exposed animals at the end of the study. Testosterone production is known to be affected by DBP (or its metabolite MBP) exposure during the MPW in rats and this has been demonstrated in studies involving exposure in-vivo, in-vitro and after xenografting^13,15,21^ and this reduction in testosterone is the proposed mechanism for the development of TDS disorders in DBP-exposed rats^8,13,22^. The relationship between DBP-induced focal testicular dysgenesis and the degree of suppression of androgens in the MPW is well established^8^. However, despite the impact of DBP exposure on androgen production in the present study there was no additional effect of DBP on inducing focal dysgenesis compared with vehicle-exposed transplants. This includes the presence of ITLCs which were identified in Sertoli cell-only cords in both vehicle- and DBP-exposed transplants. For example, there was no difference in ITLC number between the groups.

Whilst we were able to control the experiment with respect to the effect of DBP on the reaggregated xenotransplants, we acknowledge the difficulties in obtaining a directly comparable control for the focal dysgenesis identified in the vehicle-exposed reaggregated testis xenotransplants. The ‘tissue xenografts’ were obtained from testes taken at the same gestation (e17.5) as the ‘reaggregated xenotransplants’ and therefore represent a control for the grafting procedure. However, the differences in the timing of testicular development between various experimental conditions (in vivo, tissue xenografts and reaggregated xenotransplants), make it difficult to establish appropriate equivalent age-matched controls.

The reaggregation model provides a system that can be used to induce features of focal dysgenesis without a requirement for DBP exposure. This offers the advantage of avoiding chemical exposure, particularly when the mechanisms of action of DBP remain incompletely understood. In-vivo studies involving DBP exposure may also result in systemic toxicity (at higher doses) or local effects (e.g. alteration of signalling pathways) which can impact on studies aimed at understanding the mechanisms involved in the development of focal dysgenesis. However, it should be acknowledged that whilst the reaggregation model avoids the requirement for chemical exposure the process of dissociation may also impact signalling pathways within the reformed testicular tissues.

In conclusion, we have shown that seminiferous cord formation can be recapitulated during the MPW in rats and that these testis tissues display features consistent with focal testicular dysgenesis. Therefore, we propose that the re-aggregation model can be used for studies to model and manipulate seminiferous cord development during the MPW and as a novel approach to investigate the development of TDS in humans.
